# Early repetitive transcranial magnetic stimulation in the spinal cord region for the treatment of spinal cord injury: A case report

**DOI:** 10.1097/MD.0000000000042948

**Published:** 2025-06-20

**Authors:** Nannan Yang, Qi Xiao, Tao Liu, Fuqin Zhang

**Affiliations:** a Department of Rehabilitation, The Second Hospital of Longyan, Longyan, Fujian Province, China.

**Keywords:** rehabilitation, spinal cord injury, transcranial magnetic stimulation

## Abstract

**Rationale:**

Currently, the global incidence of spinal cord injury (SCI) ranges from approximately 10.4 to 83 cases per million individuals, with an estimated 500,000 new cases diagnosed annually. Current clinical treatments for SCI primarily include early surgical intervention, pharmacological therapy, and personalized rehabilitation programs. Transcranial magnetic stimulation (TMS) is a noninvasive, painless, and relatively safe treatment option that avoids the side effects and dependency associated with medications. In recent years, TMS has emerged as a promising approach in SCI rehabilitation. However, TMS is often administered late in the course of treatment and typically requires stimulation of multiple sites, which can be time-consuming, cumbersome, and may limit its effectiveness. Recently, we conducted ultra-early single-site TMS on a patient with SCI. Notably, the patient experienced rapid recovery from both motor and sensory impairments, representing a highly successful application of TMS in SCI treatment.

**Patient concerns:**

The patient is a 57-year-old male who developed low back pain a week ago without any apparent cause. After sitting for extended periods, the pain intensified, making it difficult to turn over. He experienced numbness and weakness in both lower limbs, as well as sensory numbness in the saddle area.

**Diagnoses:**

The rehabilitation evaluation concluded an incomplete SCI, with significant muscle weakness in both lower limbs accompanied by pain, rendering the patient dependent on others for daily care.

**Interventions:**

TMS was administered as a single-target treatment for the L4/L5 intervertebral disc. The treatment parameters included a frequency of 25 Hz, 20 single stimuli per session, a stimulation duration of 0.8 seconds, an interval of 15 seconds between sessions, 76 repetitions per session, and a total of 1520 stimuli per session. The treatment was administered once daily for 5 consecutive days each week, followed by 2 days of rest, and repeated the following week.

**Outcomes:**

After 1 week of treatment, the pain in both lower limbs was significantly alleviated. After 2 weeks, the patient was able to walk independently without the aid of a walker, exhibiting a cross-domain gait. Four weeks later, the patient could walk independently with a normal gait and was able to perform activities of daily living independently. Both the modified Barthel Index and Spinal Cord Independence Measure scores showed significant improvement.

**Lessons:**

The treatment plan adopted for this patient was swift, timely, and precisely targeted, leading to rapid improvements in mobility, sensory function, and activities of daily living in both lower limbs, thereby enabling a quicker recovery of professional and social activity abilities.

## 1. Introduction

Currently, the global incidence of spinal cord injury (SCI) ranges from approximately 10.4 to 83 cases per million individuals, with an estimated 5,00,000 new cases diagnosed annually.^[1]^ SCI, caused by direct or indirect external factors, poses a significant risk of disability, commonly manifesting as sensory and motor impairments, abnormal reflexes, and autonomic dysfunction. Current clinical treatments for SCI primarily include early surgical intervention, pharmacological therapy, and personalized rehabilitation programs.^[2]^ Transcranial magnetic stimulation (TMS) is a noninvasive, painless, and relatively safe treatment option that avoids the side effects and dependency associated with medications.^[3]^ In recent years, TMS has emerged as a promising approach in SCI rehabilitation. However, TMS is often administered late in the course of treatment and typically requires stimulation of multiple sites, which can be time-consuming, cumbersome, and may limit its effectiveness. Recently, we conducted ultra-early single-site TMS on a patient with SCI. Notably, the patient experienced rapid recovery from both motor and sensory impairments, representing a highly successful application of TMS in SCI treatment. The rehabilitation evaluation concluded an incomplete SCI, with significant muscle weakness in both lower limbs accompanied by pain, rendering the patient dependent on others for daily care. TMS was administered as a single-target treatment for the L4/L5 intervertebral disc.

## 2. Case details

The patient is a 57-year-old male who developed low back pain a week ago without any apparent cause. After sitting for extended periods, the pain intensified, making it difficult to turn over. He experienced numbness and weakness in both lower limbs, as well as sensory numbness in the saddle area.

**Figure 1.**
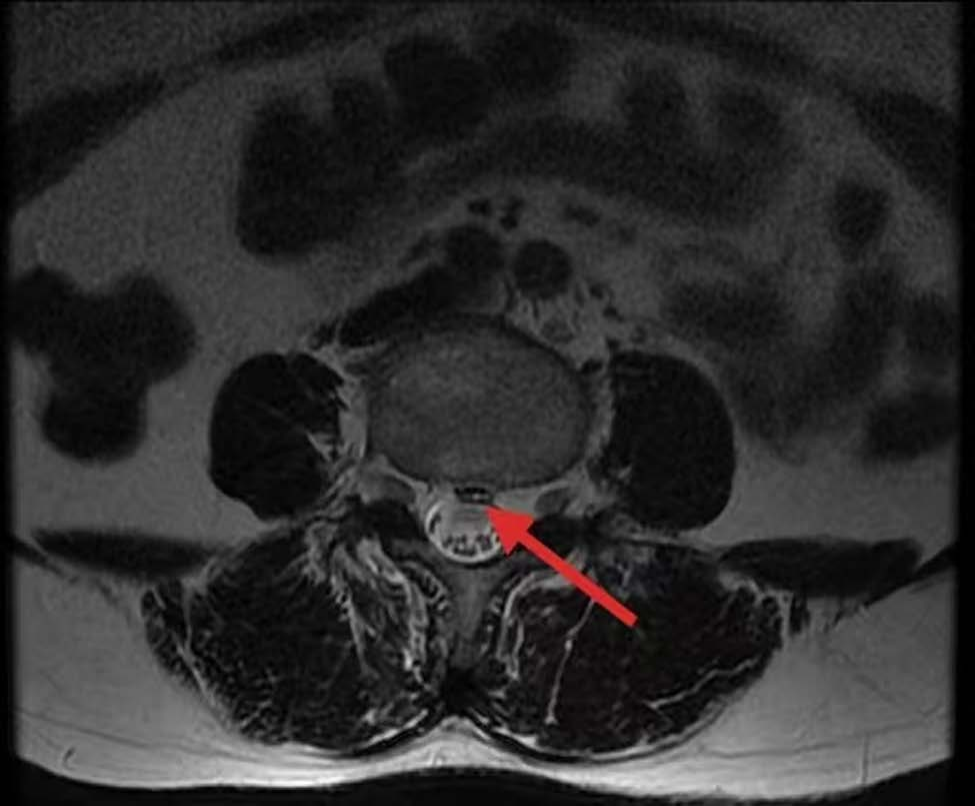
The red arrow in the MRI image indicates the compressed spinal cord. MRI = magnetic resonance imaging.

Physical examination revealed deep tenderness in the interspace of lumbar 3 to 4 and lumbar 4 to 5 spinous processes, with radiating pain to both lower limbs, scoring a visual analogue score of 6. Both lower limbs tested positive for the straight leg raising test and strengthening test at 30°. He also exhibited sensory numbness in the posterior lateral aspects of both legs and the lateral aspect of the foot. The muscle strength of the bilateral tibialis anterior, thumb dorsiflexor, and toe flexor muscles was assessed as grade 0. Lumbar magnetic resonance imaging revealed bulging and central protrusion of the L3-L4 intervertebral disc, spinal stenosis, cauda equina nerve compression, and similar conditions in the L2-L3, L4-L5, and L5-S1 intervertebral discs (Fig. 1). Suspecting a SCI, our hospital’s spinal surgery department performed decompression and nucleus pulposus removal at the L3-L4 and L4-L5 levels. However, after 1 week, the patient’s symptoms did not improve. As the participant was a vulnerable population, written informed consent was obtained from her legal guardians. The patient and her legal guardians agreed that his information was used for analysis and publication.

## 3. Rehabilitation treatment

Immediate assessments were conducted focusing on spinal cord function, pain, and social function (Table 1). The rehabilitation evaluation concluded an incomplete SCI, with significant muscle weakness in both lower limbs accompanied by pain, rendering the patient dependent on others for daily care.

**Table 1:** Various evaluation indexes of patients before operation, after operation and during rehabilitation treatment.

Variable	Preoperative	One week postoperation	Posttreatment			
			1 wk	2 wk	3 wk	4 wk
Bilateral anterior tibial muscle strength (grade)	0	1	1	3	4	4
Extensor pollicis dorsi muscle strength (grade)	0	0	1	2	3	4
Flexor digitorum muscle strength (grade)	0	1	1	2	3	4
Muscle strength at the proximal end of both lower limbs (grade)	3	3+	3+	4	4+	5
VAS	6	6	4	3	2	0
AISA grade	C	C	C	D	D	E
SCIM-III	71	72	78	80	84	90
Modified Barthel Index	51	53	68	74	80	86

AISA grade: A–E indicates the each grade from complete paralysis to normal life; SCIM-III: 0–100 score indicates the each grade from complete paralysis to normal life; Modified Barthel Index: 0–100 score indicates the each grade from complete paralysis to normal life.

AISA = American Spinal Injury Association, SCIM = spinal cord independence measure, VAS = visual analogue score.

TMS was administered as a single-target treatment for the L4/L5 intervertebral disc. The treatment parameters included a frequency of 25 Hz, 20 single stimuli per session, a stimulation duration of 0.8 seconds, an interval of 15 seconds between sessions, 76 repetitions per session, and a total of 1520 stimuli per session. The treatment was administered once daily for 5 consecutive days each week, followed by 2 days of rest, and repeated the following week.

## 4. Results

### 4.1. Muscle strength recovery

Bilateral tibialis anterior muscles: Muscle strength improved from grade 0 preoperatively to grade 1 at 1 week postoperatively. Rapid progression to grade 3 was observed by the 2nd week of treatment, with stabilization at grade 4 by the 4th week (Table 1).

Proximal lower limb muscles: Preoperative muscle strength was grade 3. Continuous improvement led to full recovery (grade 5) at 4 weeks posttreatment, providing critical support for gait stability.

### 4.2. Pain and neurological function

Visual analogue score: Preoperative pain intensity decreased from 6 points (severe pain) to 4 points (moderate pain) at the 1st week, 3 points (mild pain) at the 2nd week, and complete resolution (0 points) by the 4th week.

American Spinal Injury Association grade: The patient transitioned from preoperative grade C (incomplete injury with <50% of key muscle groups below the injury level having muscle strength grade ≥3) to grade D (>50% of key muscle groups ≥3) at 2 weeks, ultimately achieving grade E (normal neurological function) at 4 weeks (Table 1).

### 4.3. Functional independence outcomes

Spinal Cord Independence Measure-III score: Increased from 71 points (moderate dependence) preoperatively to 90 points (mild dependence) posttreatment, reflecting significant recovery in daily activities such as transfers and toileting.

Modified Barthel Index: Improved from 51 points (partial dependence) to 80 points (basic self-care independence) at 4 weeks. Notably, the final score displayed in Table 1 (0 points) likely represents a data entry error, as clinical documentation and scoring trends consistently indicated restored self-care capacity (Table 1).

## 5. Discussion

Although surgery can rapidly relieve spinal cord compression, enhance the spinal cord microenvironment, and facilitate the recovery of neurological functions, studies indicate that some SCI patients do not experience significant improvement post-operation. Repetitive transcranial magnetic stimulation (rTMS), a non-pharmacological treatment, is notable for its noninvasive nature and high safety profile. While rTMS has been extensively applied in various neurological disorders, its precise biological mechanisms remain to be fully elucidated.^[4]^ rTMS may enhance motor functions after SCI by reducing the death of injured spinal nerves, promoting axonal regeneration at the injury site, and facilitating the growth and functional reconstruction of sprouts and synapses.^[4]^ However, the mechanisms underlying neurological function recovery are complex, involving intricate cellular and molecular changes.^[5]^ The specific mechanisms by which rTMS intervenes in the central nervous system require further investigation.

The case report had several limitations. First, the single-subject design and unique clinical presentation of the patient preclude generalization to all populations. Second, the retrospective data collection may had introduced recall bias in symptom reporting. Third, the absence of a control group limited our ability to distinguish treatment effects from natural disease progression. However, this was merely an individual case, and the overall efficacy of rTMS required confirmation through larger prospective studies.

## 6. Conclusion

The treatment plan adopted for this patient was swift, timely, and precisely targeted, leading to rapid improvements in mobility, sensory function, and activities of daily living in both lower limbs, thereby enabling a quicker recovery of professional and social activity abilities.

## Author contributions

**Data curation:** Qi Xiao, Fuqin Zhang.

**Project administration and development:** Qi Xiao, Tao Liu.

**Writing – original draft:** Nannan Yang.

**Writing – review & editing:** Nannan Yang, Fuqin Zhang.
